# Relationship between physical activity and perceptions of ageing from the perspective of healthy ageing among older people with frailty with chronic disease: a cross-sectional study

**DOI:** 10.1186/s12912-023-01481-9

**Published:** 2023-09-16

**Authors:** Yu-Rung Wang, Huan-Fang Lee, Pei-Lun Hsieh, Chia-Hsiu Chang, Ching-Min Chen

**Affiliations:** 1 Department of Nursing, Chang Gung University of Science and Technology, Chiayi Campus, Taiwan; 2 Department of Nursing, College of Medicine, National Cheng Kung University, Tainan City, Taiwan; 3 Department of Nursing, College of Health, National Taichung University of Science and Technology, Taichung City, 40343 Taiwan; 4 Department of Nursing, Hungkuang University, Taichung City, 433304 Taiwan

**Keywords:** Community long-term care stations, Healthy ageing perspective, Chronic disease, Older people

## Abstract

**Background:**

In Taiwan, the 2019 Elderly Frailty Assessment found that 11.2% of older people have frailty problems. Some researchers have found that older persons’ negative perspectives on ageing aggravate the progression of frailty, thereby increasing their risk of disability. This study aimed to investigate associations of physical activity and perceptions of ageing on perspectives of healthy ageing in older people with frailty and chronic diseases and to compare the differences in their frailty status.

**Methods:**

This study used a descriptive cross-sectional design. Participants were recruited from community long-term care stations. The inclusion criteria were (1) no severe cognitive impairment and ability to communicate in Mandarin and Taiwanese; (2) over 65 years old; (3) at least one chronic disease; and (4) at least one debilitating item in the Study of Osteoporotic Fracture index. A total of 312 participants were recruited. The Brief Ageing Perceptions Questionnaire Chinese version, Healthy Ageing Perspectives Questionnaire, and Physical Activity Scale for the Elderly Chinese Version were used for measurement.

**Results:**

The study results found that demographic variables, perceptions of ageing, and physical activity were significantly correlated with perspectives on healthy ageing, including age, Activities of Daily Living, education, all domains of perceptions of ageing, and household- and work-related physical activity. With regard to the frailty status level, prefrailty was better than frailty from the perspective of healthy ageing in older people with chronic disease (*t* = 5.35, *p* < 0.05). Hierarchical regression analysis was used to predict the healthy ageing perspectives of older persons with chronic disease involving a chronic time-line, positive control, health-related changes, and work-related activities. Those domains could predict 21% of the variance in healthy ageing perspectives.

**Conclusion:**

It is suggested that in community long-term care stations, health care providers can arrange activities to improve the perception of ageing that are acceptable for older people with frailty and chronic diseases and encourage older people to participate in service activities to achieve a sense of social participation.

## Background

According to the World Health Organization [1], the older people has almost doubled over the past 35 years, from 12 to 22% of the global population, and will account for one-fifth of the global population in the near future. According to the estimation of the National Development Council [2], the older people in Taiwan is increasing each year. In 2019, a survey conducted in Taiwan revealed that 11.2% of people aged above 65 have frailty [3]. In 2022, the older people aged 65 years and above reached 17.5%, and it is estimated that by 2065, the older people will account for 41.2% of the population. Thus, Taiwan faces the problem of rapid ageing.

A report on ageing and health published by the WHO in 2015 noted that the concept of health is no longer the absence of disease, and special attention should be given to the concept of healthy ageing. The WHO healthy ageing model emphasizes the importance of influencing factors, including the social, economic, and built environments. Healthy ageing should include the management of diseases and symptoms, lifelong cognition as well as physical, mental, and social health promotion, and adjustments to social policies and clinical phenomena [4]. The WHO also stated in the Integrated Caring for Older People Guidelines (ICOPE) published in 2017 that older adults care requires health management and advice related to primary prevention and disease management, which can help the older people to improve and maintain their functioning to meet the goals of healthy ageing and independent living [5]. Regarding the impact of demographic attributes and chronic disease on healthy ageing, some studies have found that older adults are associated with decreased perceptions of health, including individuals who are older, female, low-educated, low-income, single, and living alone as well as those with chronic conditions [6–9]. Warmoth et al. found that people with negative perceptions of ageing were more prone to frailty and that these perceptions could predict frailty problems among older people in 6 years [10].

Some studies indicated that frail older people decreased physical activity levels, which may affect their perspective on healthy ageing [11, 12]. Levy et al. [13] and WHO [14] emphasized the importance of a positive perception of aging and healthy aging for older individuals. Hansen-Kyle [15] and Howse [16] mentioned that the awareness of healthy aging required preventing disability and encouraging older people’s ability to support self-health. Therefore, regular physical activity was essential for enhancing physical functioning, preventing disabilities, and actively participating in life, ultimately reducing mortality risk [14].

Given the problems of ageing and frailty, Taiwan began to promote the Long-Term Care Plan 2.0 in 2017. The scope of care was extended to primary prevention, and frail older people were included as the objects of care while promoting aging in place. The care service model of Community Long-Term Care Stations (CLTC stations) was based on The Program of All-Inclusive Care for the Elderly (PACE) from the United States [17] and Japan’s long-term care insurance system. These stations were established within local communities [18]. The CLTC stations were to provide diversified services such as shared meals or meal delivery, health promotion activities, preventive and delayed disability care services, community care visits or phone greetings, and health counselling and referrals to improve the functions of primary prevention and delay deterioration due to disability among older people in communities [19]. Based on the above, to cope with rapid ageing and the increased need for care among older people with chronic diseases and under the promotion of the Long-Term Care Plan 2.0, strategies related to preventing and delaying of disability for frail older people at CLTC stations have been conducted. However, studies at home and abroad have not explored the influence of physical activities and perspectives on healthy ageing. Therefore, this study aimed to explore the influence of physical activities and ageing perception on the perspectives of healthy ageing for frail older people with chronic diseases to serve as a reference for future policy-making and care.

## Methods

### Research design

This study involved descriptive cross-sectional research. The research period was from April 2021 to the end of March 2022. The B-APQ-C (Brief Ageing Perceptions Questionnaire Chinese version) [20], HAPQ (Healthy Ageing Perspectives Questionnaire) [21], and PASE-C (Physical Activity Scale for the Elderly Chinese version) [22] were mainly used to probe the physical activity, ageing perceptions, and healthy ageing perspectives among frail older people with chronic diseases. Therefore, this study aimed to explore the association of physical activities and ageing perception on the perspectives of healthy ageing for frail older people with chronic diseases to serve as a reference for future policy-making and care.

### Measures

The measurement tools for frail older people with chronic diseases included demographic variables and three survey scales. The demographic variables included age, gender, marital status, level of education, occupation, chronic conditions, household income, height, weight, smoking, drinking, and living patterns. The survey scales included frailty status, the Brief Ageing Perceptions Questionnaire (B-APQ) Chinese version, the Healthy Ageing Perspectives Questionnaire, and the Physical Activity Scale for the Elderly, which are detailed as follows.

1. Frailty status was assessed using the Study of Osteoporotic Fracture (SOF) to evaluate frail older people in CLTC stations [23]. To expedite the identification of frailty patients within the community, the SOF index has become widely utilized among healthcare providers. It was used to predict falls, disability, fractures, and mortality-related comorbidities [24, 25]. The scale assessment items included weight loss, lower extremities, and reduced energy level. If older adults in the community have more than two items, this is designated as “frailty.“ If they have only one item, this is designated as “prefrailty.“ The concurrent validity of the SOF index was established through a moderate to high correlation (Spearman’s rho = 0.51–0.75) with the Fried Frailty Phenotype index [23].
2. The Brief Ageing Perceptions Questionnaire (B-APQ), published by Barker in 2007, measures the two aspects of ageing perception and health-related experience. It includes 32 items and 7 dimensions on a 5-point Likert scale, with 1 indicating strongly disagree and 5 indicating strongly agree. The higher the score, the better the ageing perception [26]. Sexton et al. subsequently revised the scale to a 17-item scale with 5 dimensions, all with good validity and reliability (CFA: chi-square = 1433.54, df (109), *p* < 0.01, RMSEA = 0.04, CFI = 0.97, TLI = 0.96; Cronbach’s alpha: 0.75 ~ 0.84) [27]. Wang et al. [20] then translated it into the Chinese version in accordance with the process of translation and adaptation of instruments [28]. One localized item was added according to experts’ suggestions to make the 18-item Brief Ageing Perceptions Questionnaire Chinese version and a 23-item Health-Related Experience Questionnaire with CVIs of 0.9-1 and 0.8-1, respectively. After formal administration of the questionnaires, the Cronbach’s α of each dimension was between 0.86 and 0.91, while the overall internal consistency Cronbach’s α was 0.87. After the exploratory factor analysis and confirmatory factor analysis, the original 5 dimensions were adjusted to 4, and the overall reliability and validity of the new model and model fit were good (chi-square: 387.79, GFI: 0.91, RMSEA: 0.07, SRMR: 0.054; AGFI: 0.88) [20]. In this study, the internal consistency of Cronbach’s α was 0.82 for frail older people.
3. The Healthy Ageing Perspectives Questionnaire (HAPQ) was based on the analysis results of 3 focus group discussions and 6 qualitative in-depth interviews [21]. It also referred to a structured questionnaire based on healthy ageing theory proposed by Ryff and Singer [29] and the healthy aging model proposed by the World Health Organization. The scoring of this questionnaire was based on a 5-point Likert scale, with 1 indicating strongly disagree and 5 indicating strongly agree. The higher the score, the better the ageing perspective. The original scale had a total of 21 items, and the content validity index (CVI) was 0.8-1. After exploratory factor analysis, two items with low factor loadings were deleted. The finalized scale had a total of 19 items. The internal consistency of each dimension in the formal testing was between 0.71 and 0.89, while the overall Cronbach’s α internal consistency was 0.80. The overall scale explained 62.71% of the healthy ageing status among older adults with chronic diseases. The questionnaire established criterion validity through a significant correlation (ranging from 0.14 to 0.65) with the World Health Organization Quality of Life scale (WHOQOL) [21]. For our study, the internal consistency of Cronbach’s α was 0.71 for frail older people.
4. The Physical Activity Scale for the Elderly used the Chinese version of the Physical Activity Scale (PASE-C) published by Ku et al. [22]. This is a 12-item scale focusing on the physical activities of older people in their lives, including the overall activity level, leisure, household, and work-related physical activities. The questionnaire was tested for reliability and validity and was simultaneously measured with accelerometer measures (energy expenditure and walking steps), grip strength, and the Geriatric Depression Scale (GDS) for reliability and validity. The intraclass correlation (ICC) of the test-retest was 0.85. In terms of concurrent validity, the total score of the male and female scales showed a significantly positive correlation with grip strength (*r* = 0.34 for males, *r* = 0.24 for females) and a significantly negative correlation with the Geriatric Depression Scale (GDS) (*r* = 0.38 for total score). The reliability and validity test results were supported by evidence. Our study’s criterion validity with the overall physical activity and B-APQ showed a significantly positive correlation (r = 0.39).

### Participants

The participation criteria followed the Study of Osteoporotic Fracture (SOF) criteria for the assessment of frailty in older people [30] in the CLTC stations under the Long-Term Care Plan 2.0 of the Ministry of Health and Welfare in Taiwan. The inclusion criteria included (1) the ability to communicate in Mandarin Chinese and Taiwanese; (2) aged 65 years or above; (3) at least one chronic according to the WHO definition [31]; and (4) meeting at least one item of the frailty index in the SOF frailty assessment results. The exclusion criteria were cognitive impairment and inability to communicate verbally. G*power 3.1.9.2 was used to calculate the sample size. The type I error α was set at 0.05, the power at 0.8, and the explanatory power R^2^ was a medium effect size (0.13). The number of samples required was 260. With the estimated sample attrition rate of 20%, a total of 312 people were needed [32].

### Data collection

Before data collection, the researcher first contacted the competent authority of the CLTC stations in the community care centres and entered the care centres to collect data after obtaining consent. The purpose of this study was explained to the older people in the care centres, and those who were not willing to be interviewed were told that their decision would not impact their rights and interests in the care centres. Those who were willing to participate in the study signed consent forms before the 20- to 30-minute face-to-face interviews with the researcher or the research assistant coupled with the questionnaire survey. After collecting the data, the participating elders were given a small gift. Before collecting the questionnaire, the inter-rater reliability (Kappa coefficient) was conducted to examine the consistency between the researcher and the research assistant, the Kappa coefficient was 0.9 in the current study.

### Data analysis

The collected data were organized and analysed using SPSS 23.0 for Windows. The demographic variables were analysed by means, standard deviation (SD), and percentages. The association of demographic characteristics, physical activity level, and aging perceptions with healthy ageing perspectives were further analysed by independent t-test, Pearson product-moment correlation, and one-way analysis of variance (ANOVA). Finally, hierarchical regression analysis was used to explore the impact of the significant variables in demographic characteristics, level of physical activity, and ageing perceptions on healthy ageing perspectives among frail older people with chronic diseases.

## Results

### Demographic characteristics

This study included 312 frail older people with chronic diseases in Taiwan, whose average age was 75.71 years (*SD* = 7.84), with females accounting for the majority (69.9%). They had at least 2 chronic diseases (*SD* = 0.69), with hypertension being the most common chronic disease (63.14%), followed by diabetes (17.95%) (Table 1). For the remaining demographic variables, most of the older adults were overweight (59.9%) and in the stage of prefrailty (52.9%), had an education level of junior high school and below (83%), had religious beliefs (95.8%), were not working (76%), were married (54.2%), had a monthly household income of NT$ 20,000 and below (68.3%), were nonsmokers (95.8%), were nondrinkers (98.1%), and lived with their children (55.1%) (Table 1).

**Table 1:** Demographic characteristics of the participants and associated with the perspectives on healthy aging

Items	N	%	Mean	SD	*r*	*F*	*t*	*p* value
**Age**			75.71	7.84	-0.24			< 0.001
**Chronic conditions**			1.45	0.69	-0.06			0.296
Hypertension	197	63.14%						
Diabetes	56	17.95%						
Hyperlipidemia	38	12.18%						
Cardiovascular disease	45	14.42%						
Osteoarthritis	43	13.78%						
Digestive diseases	23	7.37%						
Urinary system disease	12	3.85%						
Stroke	11	3.53%						
Nervous system disease	8	2.56%						
Lung disease	6	1.92%						
Other	8	2.56%						
**BMI**						1.41		0.247
Underweight (< 18.5)	6	1.9%						
Healthy Weight (18.5 ~ 24)	119	38.1%						
Overweight (> 24)	187	59.9%						
**ADL**			90.05	7.84	0.30			< 0.001
**Frailty index**								
Pre-frailty	165	52.9%					5.35	< 0.001
Frailty	147	47.1%						
**Gender**								
Female	218	69.9%					-0.21	0.833
Male	94	30.1%						
**Education**								
Junior high school & below	259	83.0%					-3.02	0.003
Senior school & above	53	17.0%						
**Occupation**								
Yes	75	24.0%					-0.49	0.627
No	237	76.0%						
**Religion**								
Yes	299	95.8%					0.44	0.663
No	13	4.20%						
**Marital status**								
Single/ Widowed/ Divorced	143	45.8%					-2.36	0.019
Married	169	54.2%						
**Household income**								
NT 20,000 & below (Low income)	213	68.3%					-0.63	0.533
NT 20,000 & above	99	31.7%						
**Smoking**								
Yes	13	4.20%					-0.18	0.854
No	299	95.8%						
**Drinking**								
Yes	6	1.90%					-0.11	0.911
No	306	98.1%						
**Living patterns**								
Children	172	55.1%						
Spouse	86	27.6%				2.96		0.053
Living alone	54	17.3%						

### Correlation between B-APQ, PASE-C and perspectives on healthy aging

Among the demographic variables of frail older people with chronic diseases, those with older age (*r* = -0.24, *p* < 0.05), with low education level (*t* = -3.02, *p* < 0.05), and who were single/widowed/divorced (*t* = -3.36, *p* < 0.05) had worse healthy ageing perspectives, while those with better physical activity (*r* = 0.30, *p* < 0.05) had better healthy ageing perspectives, showing a significant difference (Table 1). In particular, the frailty index showed that older persons with chronic disease had better prefrailty (mean = 3.22, *SD* = 0.25) than frailty (mean = 3.08, *SD* = 0.21) from a healthy ageing perspective (*t* = 5.35, *p* < 0.05). In terms of ageing perception, the better the scores in *time-line chronic* (*r* = 0.21, *p* < 0.05), positive control (*r* = 0.17, *p* < 0.05), and *negative ageing control emotion and consequences* (*r* = 0.26, *p* < 0.05), the better their healthy ageing perspectives. For health-related changes, the higher the scores in *health-related changes* (*r* = -0.32, *p* < 0.05) and *health-related changes due to ageing* (*r* = -0.32, *p* < 0.05), the worse their healthy ageing perspectives. For the Physical Activity Scale for the Elderly (PASE-C), the higher the scores in overall activity (*r* = 0.22, *p* < 0.05), leisure activity (*r* = 0.11, *p* < 0.05), household activity (*r* = 0.21, *p* < 0.05) and work activity (*r* = 0.30, *p* < 0.05), the better their healthy ageing perspectives (Table 2).

**Table 2:** Correlation between B-APQ, PASE-C and perspectives on healthy aging

Item	Mean	SD	*r*	
**Aging perceptions (B-APQ)**				
Time-line chronic	2.15	0.77	0.29^***^	
Consequences positive	3.55	0.59	0.11	
Control positive	3.62	0.55	0.17^**^	
Negative aging control emotion and consequences	2.84	0.58	0.26^***^	
**Health related changes (HRC)**				
Health related changes	8.38	5.49	-0.32^***^	
Health related changes due to aging	8.21	5.50	-0.32^***^	
**Physical Activity Scale for the Elderly (PASE-C)**				
Leisure	19.86	31.74	0.11^*^	
Household	47.53	35.10	0.21^***^	
Work	1.17	2.52	0.30^***^	
Overall	68.57	53.69	0.22^***^	
**Healthy aging perspectives questionnaire (HAPQ)**				
Physical and psychological changes caused by disease	2.69	0.60	0.41^***^	
Social relationship changes caused by disease	3.30	0.47	0.60^***^	
Lifestyle created by the disease	3.23	0.46	0.49^***^	
Changes in family life caused by disease	3.54	0.40	0.52^***^	
Total score	3.15	0.24	1	

Note. *p ≦ 0.05 ** *p* ≦ 0.01; ***p ≦ 0.001

### Results of hierarchical regression analysis for healthy aging

In the establishment of the Model 1 regression model of frail older people with chronic diseases, since ADL was highly correlated with frail status (*r* = 0.70, *p* < 0.05), the regression model was entered with the frailty condition. Frailty was one significant variable in Mode 1, with *R*^*2*^ = 0.10; after adjustment, *R*^*2*^ = 0.09 and *F* value = 8.83 (*p* < 0.05). Step 2 added the ageing perception (B-APQ-C) and health-related changes (HRC) for the establishment of the Mode 2 regression model. Since *health-related changes* were highly correlated with *health-related changes due to ageing* (*r* = 0.99, *p* < 0.05), *health-related changes* were selected into the analysis of the regression model. The results showed that the variables with significant influence were *time-line chronic, positive control*, and *health-related changes*, with *R*^*2*^ = 0.22; after adjustment, the *R*^*2*^ = 0.19 and the *F* value = 10.35 (*p* < 0.05), and the explanatory power increased by 12% compared with Mode 1. Step 3 added the leisure, household, and work activities of the PASE-C for the establishment of the Mode 3 regression model. The results showed that the variables with significant influence were *time-line chronic, positive control, health-related changes*, and work activities, with *R*^*2*^ = 0.24; after adjustment, *R*^*2*^ = 0.21 and the *F* value = 8.61 (*p* < 0.05) (Table 3).

**Table 3:** Results of hierarchical regression analysis for healthy aging

Variable	Model 1	Model 2	Model 3
	β	β	β
Step 1: Demographic			
Age	-0.08	0.03	0.06
Frailty (Ref: Pre-frailty)	-0.22^*^	-0.12	-0.11
Education (Ref: Junior high school & below)	0.09	0.06	0.06
Marital status (Ref: Single/ Widowed/ Divorced)	0.04	0.04	0.06
Step 2: B-APQ-C and HRC			
B-APQ-C			
Time-line chronic		0.18^*^	0.14^*^
Control positive		0.27^***^	0.24^***^
Negative aging control emotion and consequences		0.11	0.10
Health related changes (HRC)		-0.15^*^	-0.16^*^
Step 3: PASE-C			
Leisure			0.01
Household			-0.03
Work			0.18^**^
R^2^	0.10^***^	0.22^***^	0.24*
Adjusted R^2^	0.09	0.19	0.21
F	8.83^***^	10.35^***^	8.61^***^

Note. *p ≦ 0.05 ** *p* ≦ 0.01; ***p ≦ 0.001

## Discussion

This study explored associations of ageing perception and physical activities on healthy ageing perspectives among older people with frailty with chronic diseases. The subjects were frail older adults with chronic diseases in the Community long-term care stations established under the Long-Term Care Plan 2.0. The study’s participants were close to the average age of 75 and above and were the subjects of the Long-Term Care Plan 2.0 of the Ministry of Health and Welfare in 2021 [33]. Our study, similar to the Japanese government’s report on an aging society, found that frail older adults over the age of 75 in Japan require nursing care in the community [34]. In terms of health data, lifestyle and other conditions, the BMI results of the participants in this study were different from the average of the results in the 2013–2016 Nutrition and Health Survey in Taiwan (NAHSIT) [35]. The BMI of the participants in the present study showed that most were overweight. The main reason was that the participants in this study were mainly frail older adults with chronic diseases in the community, in contrast to the Nutrition and Health Survey, whose participants were not limited to frail older adults with chronic diseases [35]. The results of our study are consistent with those of a systematic review, which found that obesity is one of the risk factors in frail older people [36]. Furthermore, regarding smoking and drinking behaviours, the results of this study showed that the participants had lower rates of smoking and drinking compared to those found in surveys conducted in China and Japan [37, 38]. The difference could be associated with the higher average age of the participants, which may also lead to a lower rate of poor lifestyle. In addition, in terms of the living patterns among older people, most older people in Taiwan live with their children (55.1%) and most of them are families with three generations living together. This finding is similar to the results of the Report of the Senior Citizen Condition Survey 2017, where 54.3% of the older people surveyed expected to live with their children [39]. Taiwan’s family construct was the majority of three-generation households and different from nuclear families in China [40].

To date, only a few studies have mentioned the factors that affect healthy ageing [41–43]. Therefore, we compared studies that recruited the same group as the subjects and had similar concepts, such as self-perceived health status, quality of life (including mental and physical), and well-being. The study found that the older people’s age, the worse their physical activity and the greater their level of frailty, which lowers health ageing perspectives among frail older people with chronic diseases. It has also been suggested in several studies that the older people’s age is, the greater their decline in perceived health status [6, 7, 44]. The level of education also affects heath ageing perspectives. People with a higher level of education had better self-perceived heath, and those whose marital status is single/widowed/divorced had lower scores in healthy ageing than those who were married [6, 7, 43], similar to the findings in this study.

The results of the study showed that ageing perceptions (B-APQ-C) and the results of the subscales of health-related changes were significantly correlated with healthy ageing perspectives. The influence of positive ageing perspectives in older people’s lives improves their well-being [27]. This finding is similar to the results of a related Taiwanese study that showed that the more positive people’s attitude towards ageing was, the better their overall health status was in a self-assessment [45]. A British study also noted that an optimistic ageing attitude was better for self-perceived health status [46], and a study conducted by Levy et al. showed that the more positive older people’s ageing perceptions were, the better their quality of life and well-being [47].

In terms of associations of physical activity on healthy ageing, leisure activity, household activity, and work activity had significantly low correlations with healthy ageing perspectives among frail older people with chronic diseases. Compared with the healthy ageing perspective of older people with chronic diseases [41], this study found that some frail older people with chronic diseases still thought that they were able to work, which had a positive association with their healthy ageing perspectives. The results are also similar to an Australian study on the total scores of the Health Ageing Quiz and the Frequency Activities Index, which showed that higher activity frequency promoted healthy ageing and optimistic attitudes [42].

When predicting the healthy ageing perspectives of frail older people, the demographic variable with the greatest influence was the degree of frailty. After adding the perspectives of ageing perception, the explanatory power gradually increased, confirming that ageing perception had an impact on healthy ageing perspectives among frail older people with chronic diseases. It was proposed in the literature that more positive ageing perceptions improve self-perceived health, well-being and quality of life among older people [27, 48, 49]. The finding that *time-line chronic* and positive control affect healthy ageing perspectives, similar to the result of the positive impact on the quality of life in the psychological domain [48] and a greater influence on well-being [49, 50], showed that if older people have higher self-control and better management of the ageing process, their healthy ageing perspectives improve. With regard to physiological ageing-related health changes, frail older people with chronic diseases believed that the more health-related changes they experienced, the lower their healthy ageing perspectives were, similar to the results in the studies conducted by Machón and Steptoe [7, 46] showing that the more self-perceived diseases older people had, the worse their self-perceived health was. For physical activity, frail older people with chronic diseases believed that being able to conduct work activities influenced their ageing perspectives. Work activities include work for pay or as a volunteer. This may be due to the long-standing concept of exercising for health and the promotion of policies in Taiwan such as social participation, civic participation, and employment of the Healthy Ageing and Age-Friendly City Policy [51], which also affect healthy ageing perspectives.

Limitation.

The limitations of this study were that the subjects of the study were mainly members of frail groups with better mobility in the CLTC stations in Southern Taiwan, while those with poor mobility were excluded. Regarding data collection, there were fewer male participants and the research period coincided with the epidemic period. There is a possibility that this study may have been impacted by reduced activity, which could be a significant limitation of the research. In addition, a face-to-face interview questionnaire was conducted, and frail older people had to recall their physical activity for the past week with a short period of time. Therefore, there is a possibility of some recall bias, which may also cause underestimation of the activity.

## Conclusion

This study found that the time-line chronic and positive control of the ageing perspective (B-APQ-C), health-related changes, and work activity influence healthy ageing perspectives among frail older people with chronic diseases, with 24% of the explanatory power from the model. he explanatory power of ageing perceptions could be increased to 12%, showing that positive control in ageing perceptions and health-related changes had a greater association on healthy ageing.

### Implications of the study findings for community care

In the CLTC stations, when the conditions of frail older people with chronic diseases are debilitating, some work activities that they can accomplish independently within the care stations could be combined to enhance positive control, such as the cultivation of senior volunteers to enable them to have a sense of social participation and the opportunity to serve society to improve their healthy ageing perspectives further. Future studies could focus on intervention measures to enhance positive aging perceptions and physical activity among older people. Additionally, conducting long-term follow-ups would allow for observing changes in older adults following such interventions.

## Data Availability

The datasets used and/or analysed during the current study are available from the corresponding author on reasonable request.

## Abbreviations

CLTC Stations: Community Long-Term Care Stations
SOF: Study of Osteoporotic Fractures
B-APQ-C: Brief Ageing Perceptions Questionnaire Chinese version
HAPQ: Healthy Ageing Perspectives Questionnaire
PASE-C: Physical Activity Scale for the Elderly Chinese Version
BMI: Body Mass Index
ADL: Activities of Daily Living

## References

[1] Ageing and health. https://www.who.int/news-room/fact-sheets/detail/ageing-and-health.

[2] Population by five years groups. https://pop-proj.ndc.gov.tw/main_en/dataSearch5.aspx?uid=78&pid=78

[3] 2019 Elderly Frailty Assessment Report. https://data.gov.tw/dataset/128905.

[4] Aronson L (2020). Healthy aging across the Stages of Old Age. Clin Geriatr Med.

[5] Integrated Caring for Older People Guidelines. https://www.who.int/ageing/publications/guidelines-icope/en/.

[6] McLaughlin SJ, Jette AM, Connell CM (2012). An examination of healthy aging across a conceptual continuum: prevalence estimates, demographic patterns, and validity. J Gerontol A Biol Sci Med Sci.

[7] Machón M, Vergara I, Dorronsoro M, Vrotsou K, Larrañaga I (2016). Self-perceived health in functionally independent older people: associated factors. BMC Geriatr.

[8] Cramm JM, Nieboer AP (2018). Aging perceptions matter for the well-being of elderly turkish migrants, especially among the chronically ill. BMC Geriatr.

[9] Chang MY, Chang YC, Hsu SW (2012). Relationships between physical health status and medical services utilization among Taiwan’s elderly people. Taiwan J Gerontological Health Res.

[10] Warmoth K, Tarrant M, Abraham C, Lang IA (2018). Relationship between perceptions of ageing and frailty in English older adults. Psychol Health Med.

[11] Aday RH, Wallace JB (2015). Development and validation of the healthy aging incentives Scale. Educ Gerontol.

[12] Sialino LD, Wijnhoven HAH, van Oostrom SH, Picavet HSJ, Verschuren WMM, Visser M, Vader S, Schaap LA (2023). Perspectives of older women in the Netherlands: identifying motivators and barriers for healthy lifestyles and determinants of healthy aging. BMC Public Health.

[13] Levy BR, Slade MD, Kunkel SR, Kasl SV (2002). Longevity increased by positive self-perceptions of aging. J Pers Soc Psychol.

[14] World report on aging and health. http://apps.who.int/iris/bitstream/10665/186463/1/9789240694811_eng.pdf?ua=1.

[15] Hansen-Kyle L (2005). A concept analysis of healthy aging. Nurs Forum.

[16] Howse K (2012). Healthy ageing: the role of health care services. Perspect Public Health.

[17] Program of All-Inclusive Care for the Elderly (PACE). https://www.cms.gov/Medicare-Medicaid-Coordination/Medicare-and-Medicaid-Coordination/Medicare-Medicaid-Coordination-Office/PACE/PACE

[18] Status report on Long-term Care Insurance. https://www.mhlw.go.jp/topics/kaigo/osirase/jigyo/m14/1409.html.

[19] Hsu HC, Chen CF (2019). LTC 2.0: the 2017 reform of home- and community-based long-term care in Taiwan. Health Policy.

[20] Wang YR, Lee HF, Chen CM (2021). Validating a brief aging perception questionnaire (B-APQ) for older persons with chronic disease in Taiwan. Aging Ment Health.

[21] Wang Y-R, Lee H-F, Hsieh P-L, Chen C-M (2023). Development of the healthy aging perspectives questionnaire among older adults with chronic disease in Taiwan. Health Soc Care Commun.

[22] Ku PW, Sun WJ, Chang CY, Chen LJ (2013). Reliability and validity of the Chinese Version of the physical activity scale for the Elderly. Sports & Exercise Research.

[23] Hu BY, Yu HW, Chiu TY, Lin LL, Chen DR, Chen YM (2019). Validity of the study of osteoporotic Fractures Index (SOF Index): cases of community-dwelling older adults and older adults living alone. Taiwan J Public Health.

[24] Ensrud KE, Ewing SK, Taylor BC, Fink HA, Cawthon PM, Stone KL, Hillier TA, Cauley JA, Hochberg MC, Rodondi N (2008). Comparison of 2 frailty indexes for prediction of falls, disability, fractures, and death in older women. Arch Intern Med.

[25] Ensrud KE, Ewing SK, Cawthon PM, Fink HA, Taylor BC, Cauley JA, Dam TT, Marshall LM, Orwoll ES, Cummings SR (2009). A comparison of frailty indexes for the prediction of falls, disability, fractures, and mortality in older men. J Am Geriatr Soc.

[26] Barker M, O’Hanlon A, McGee HM, Hickey A, Conroy RM (2007). Cross-sectional validation of the aging perceptions questionnaire: a multidimensional instrument for assessing self-perceptions of aging. BMC Geriatr.

[27] Sexton E, King-Kallimanis BL, Morgan K, McGee H (2014). Development of the brief ageing perceptions questionnaire (B-APQ): a confirmatory factor analysis approach to item reduction. BMC Geriatr.

[28] Process of translation and adaptation of instruments. http://www.who.int/substance_abuse/research_tools/translation/en/.

[29] Ryff CD, Singer BH (2009). Understanding healthy aging: key components and their integration.

[30] Luciani A, Dottorini L, Battisti N, Bertuzzi C, Caldiera S, Floriani I, Zonato S, Ferrari D, Foa P (2013). Screening elderly cancer patients for disabilities: evaluation of study of osteoporotic fractures (SOF) index and comprehensive geriatric assessment (CGA). Ann Oncol.

[31] Noncommunicable diseases https://www.who.int/news-room/fact-sheets/detail/noncommunicable-diseases.

[32] Cohen J (1998). Statistical power analysis for the behavioral Sciences.

[33] Long term care 2.0. : demographic characteristics of long-term care service users https://www.mohw.gov.tw/dl-68885-7e5d51eb-fb9c-41d7-8208-9c05a8c93cbd.html.

[34] Annual report on the ageing society. https://www8.cao.go.jp/kourei/english/annualreport/2021/pdf/2021.pdf.

[35] Wong TJ, Yu T (2022). Trends in the distribution of body mass index, waist circumference and prevalence of obesity among taiwanese adults, 1993–2016. PLoS ONE.

[36] Kaskirbayeva D, West R, Jaafari H, King N, Howdon D, Shuweihdi F, Clegg A, Nikolova S (2023). Progression of frailty as measured by a cumulative deficit index: a systematic review. Ageing Res Rev.

[37] Okui T (2020). An age-period-cohort analysis of the difference in smoking prevalence between urban and non-urban areas in Japan (2004–2019). Epidemiol Health.

[38] Global Adult Tobacco Survey (GATS)- Fact sheet China. 2018. https://www.who.int/docs/default-source/wpro-documents/countries/china/2018-gats-china-factsheet-cn-en.pdf.

[39] Report the senior citicen condition. 2017 https://www.mohw.gov.tw/dl-70609-64499d44-6d1f-408e-a449-6af459b7cd17.html.

[40] China country assessment report on ageing and health. https://www.who.int/publications/i/item/9789241509312.

[41] Wang YR. Comparisons of the Effects of Physical Activity and Perceptions of Aging on the Perspective of Healthy Aging among Older Persons with Chronic Disease Living on Cross-Taiwan Strait. [Unpublished doctoral dissertation]. National Cheng Kung University; 2020.

[42] Cyarto EV, Dow B, Vrantsidis F, Meyer C (2013). Promoting healthy ageing: development of the healthy ageing quiz. Australas J Ageing.

[43] Manasatchakun P, Chotiga P, Hochwalder J, Roxberg A, Sandborgh M, Asp M (2016). Factors Associated with healthy aging among older persons in northeastern Thailand. J Cross Cult Gerontol.

[44] Jaspers L, Schoufour JD, Erler NS, Darweesh SK, Portegies ML, Sedaghat S, Lahousse L, Brusselle GG, Stricker BH, Tiemeier H (2017). Development of a healthy aging score in the Population-Based Rotterdam Study: evaluating age and sex differences. J Am Med Dir Assoc.

[45] Wu CH, Chen CY, Hsu CC, Wu YC (2016). Development and Psychometric Properties of the Taiwan attitude toward Aging Questionnaire (TAAQ). Formosa J Mental Health.

[46] Steptoe A, Wright C, Kunz-Ebrecht SR, Iliffe S (2006). Dispositional optimism and health behaviour in community-dwelling older people: Associations with healthy ageing. Br J Health Psychol.

[47] Levy BR, Slade MD, Kunkel SR, Kasl SV (2002). Longevity increased by positive self-perceptions of aging. J Personal Soc Psychol.

[48] Gu R, Zhang D, Jin X, Wu W, Hou Y, Wu Q, Wang X (2019). The self-perceptions of aging were an important factor associated with the quality of life in chinese elderly with hypertension. Psychogeriatrics.

[49] Slotman A, Cramm JM, Nieboer AP (2017). Validation of the aging perceptions Questionnaire Short on a sample of community-dwelling turkish elderly migrants. Health Qual Life Outcomes.

[50] Cramm JM, Nieboer AP (2018). The importance of health behaviours and especially broader self-management abilities for older turkish immigrants. Eur J Public Health.

[51] Healthy. aging https://www.hpa.gov.tw/4105/el.

